# Exploring the causal relationship between female reproductive traits and frailty: a two-sample mendelian randomization study

**DOI:** 10.3389/fphys.2024.1349952

**Published:** 2024-03-28

**Authors:** Maoxia Fan, Dandan Wang, Xiaoqi Wu, Wulin Gao

**Affiliations:** ^1^ Shandong University of Traditional Chinese Medicine, Jinan, Shandong Province, China; ^2^ Department of Geriatric Medicine, Affiliated Hospital of Shandong University of Traditional Chinese Medicine, Jinan, Shandong Province, China

**Keywords:** age at menarche, age at first birth, age at first sexual intercourse, age at natural menopause, pregnancy abortion, body mass index, educational attainment, frailty

## Abstract

**Background:** The impact of female reproductive factors, including age at menarche (AAM), age at first birth (AFB), age at first sexual intercourse (AFS), age at natural menopause (ANM), and pregnancy abortion (PA), on the risk of developing frailty remains uncertain. Our objective is to examine the potential causal relationship between female reproductive traits and frailty through the utilization of two-sample univariable Mendelian Randomization (UVMR) and multivariable Mendelian Randomization (MVMR) analyses.

**Methods:** Leveraging large-scale Genome-Wide Association Study (GWAS) data from individuals of European ancestry, we performed two-sample UVMR and MVMR analyses to examine the causal relationship between female reproductive traits and frailty. The primary analysis employed inverse-variance-weighted (IVW) estimation, and sensitivity analyses were conducted to assess the robustness of the findings.

**Results:** The UVMR analysis revealed a significant causal relationship between female reproductive traits (AFS, AFB, AAM) and frailty [IVW: OR = 0.74, 95%CI(0.70–0.79), *p* = 0.000; OR = 0.93, 95%CI(0.92–0.95), *p* = 0.000; OR = 0.96, 95%CI(0.95–0.98), *p* = 0.000]. However, there was no significant effect of ANM and PA on frailty (*p* > 0.05). The sensitivity analysis results were robust, supporting the findings. Furthermore, this association remained significant even after adjusting for body mass index (BMI) and educational attainment (EA) in the MVMR analysis [IVW: OR = 0.94, 95%Cl (0.91–0.97), *p* = 0.000; OR = 0.77, 95%Cl (0.70–0.86), *p* = 0.000; OR = 0.95, 95%Cl (0.94–0.97), *p* = 0.000]. BMI and EA serve as mediators in this process.

**Conclusion:** Our research has established a significant causal relationship between female reproductive traits (AFS, AFB, AAM) and frailty, with BMI and EA acting as mediating factors in this process. However, further research is warranted to validate our findings and elucidate the underlying biological mechanisms.

## 1 Introduction

Frailty is an age-related syndrome characterized by increased vulnerability of the body due to degenerative changes and various chronic diseases. It is defined as a clinical state in which the physiological reserve function of older adults decreases, leading to increased vulnerability and reduced ability to cope with daily or acute stress (Morley et al., 2013; Hoogendijk et al., 2019). Frailty encompasses a range of symptoms resulting from the cumulative decline in multiple physiological systems. Clinical manifestations include low physical activity, reduced body mass, fatigue, and impaired physical function (such as slow walking, decreased grip strength, and weakened muscle strength), often associated with aging. It is a complex outcome of dysregulation and dysfunction in various systems, including muscle, nerve, endocrine, and immune systems. The pathological mechanism of frailty remains incompletely understood and may involve multiple risk factors (Tsutsumimoto et al., 2018). Frail elderly individuals experience an imbalance in multiple physiological systems and a decline in physiological reserve capacity, leading to an elevated risk of falls, disability, hospitalization, and mortality. Additionally, their demand for social care and medical resources significantly rises, thereby imposing a substantial economic burden on both society and families (Hoogendijk et al., 2019).

Research has demonstrated that hormone levels in the human body undergo changes during aging and disease, exerting regulatory effects on the physiological and pathological changes of the body through the hypothalamic-pituitary axis and various endocrine organs. The intricate interrelationships between the endocrine system, brain, immune system, and skeletal muscle are regarded as one of the key systems contributing to frailty (Clegg and Hassan-Smith, 2018). Research has revealed that alterations in hormone levels, encompassing vitamin D, dehydroepiandrosterone sulfate, testosterone, growth hormone, insulin-like growth factor-1, parathyroid hormone, cortisol, estradiol, and others, play a pivotal role in the pathogenesis of frailty (Travison et al., 2011; Morley and Malmstrom, 2013). Studies have revealed that these hormones play a significant role in influencing fatigue, muscle strength, and the decline of muscle mass in the frailty phenotype. The reduction in muscle mass and strength is a crucial factor contributing to the deterioration of physical function and self-care ability. Estrogen also plays a crucial role in maintaining and regulating the integrity of cognitive-related brain networks, particularly in the hippocampus and associated structures (Dieni et al., 2018). Additionally, it can elevate the level of second messengers and facilitate cell proliferation. Estrogen deficiency decreases estrogen receptors α and promotes apoptosis of hippocampal cells, resulting in learning and memory disorders. This condition also heightens the risk of bodily harm (Kaidah et al., 2016; Pandey et al., 2020).

Female reproductive characteristics may have a significant sex-specific impact on frailty. The onset of the first menstrual period is a notable indication of puberty and signifies the initiation of a woman’s fertility. The rise in gonadotropins during adolescence can stimulate the ovaries to produce estradiol, initiating a cascade of significant physical, emotional, cognitive, and social changes in women. This hormonal event during adolescence subsequently establishes the neural circuitry underlying adult reproductive behavior and regulates neuronal morphology, quantity, and function (Jett et al., 2022; Wang et al., 2023). During the reproductive stage, the circulating levels and concentrations of estrogen in women fluctuate throughout the menstrual cycle in response to significant reproductive events. Overall, estrogen level variations throughout a woman’s lifetime are associated with multiple reproductive factors, such as menarche, first sexual activity, pregnancy, and menopause. In this context, it is hypothesized that female reproductive traits, such as age at menarche (AAM), age at first birth (AFB), age at first sexual intercourse (AFS), age at natural menopause (ANM), and pregnancy abortion (PA), may potentially contribute to an increased risk of developing frailty in women. However, the relationship between female reproductive traits and frailty remains unclear.

Mendelian Randomization (MR) analysis is an epidemiological research method that assesses the causal relationship between an exposure and outcomes by leveraging one or more genetic variations, such as single nucleotide polymorphisms (SNPs) (Davies et al., 2018). MR research can effectively mitigate the impact of reverse causal relationships and confounding factors that are prevalent in traditional epidemiological studies (Qin et al., 2006). Therefore, based on the aforementioned advantages of MR analysis, this study aims to perform univariate and multivariate MR analysis utilizing published Genome-Wide Association Study (GWAS) data. The objective is to investigate the causal relationship between female reproductive traits and frailty, as well as the potential mediation by mediators through a two-step MR analysis.

## 2 Materials and methods

### 2.1 Data sources

This study utilized the GWAS dataset to perform a two-sample Mendelian Randomization analysis, aiming to assess the causal relationship between female reproductive traits and frailty. In this study, the exposure factors consisted of female reproductive traits, namely, age at menarche (AAM), age at natural menopause (ANM), age at first birth (AFB), age at first sexual intercourse (AFS), and pregnancy abortion (PA). The outcome of interest was the occurrence of frailty. Additionally, a mediating effect analysis was performed to assess the proportion of mediating effects contributed by body mass index (BMI) and educational attainment (EA) in the relationship between female reproductive traits and frailty. The introduction of each data source is provided in Table 1. The GWAS instrumental variables (IVs) utilized in the MR analysis are presented in Supplementary Table S1-S10.

**TABLE 1:** Data sources used in the MR analyses for this study.

Pheaotyp	ID	Sample size	Number of SNPs	Category	Youth:alp	Sources
Exposures						
AAM	ebi-a-GCST9 0029036	279,470	11,971,701	Continuo	European	UK Biobank. 23andMe and ReproOn
AFS	ebi-a-GCST9 0000047	397,338	16,359,424	COMiD110111S	European	UK Biobank
AFB	ebi-a-GCST9 0000050	542,901	9,702,772	Continuous	European	UK Biobank
ANM	ukb-b-17422	143,819	9,851,867	Continuous	European	UK Biobank
PA	finn-b-015_P REG ABOR T	123,579 (14case:34,239 NcontroL89,34,0)	16,379,784	Binary	European	FinnGen
Outcomes						
Frailty	ebi-a-GCST9 0020053	175,226	7,589,717	Binary	European	UK Biobank, TuunGene and SATSA
Mediations						
EA	ebi-a-GCST9 0029013	461,457	11972.619	Continuous	European	UK, Biobank
BMI	ieu-b-40	681,275	2,336,260	Continuous	European	UK, Biobank

Note: AAM, age at menarche; AFB, age at first birth; AFS, age at first sexual intercourse; ANM, age at natural menopause; PA, pregnancy abortion; BMI, body mass index; EA, educational attainment; MR, mendelian randomization.

### 2.2 Selection of IVs

MR research must adhere to the three fundamental assumptions of relevance, independence, and exclusivity, which are as follows: ① IVs should exhibit a strong correlation with the exposure factors; ② IVs should not be associated with any confounding factors related to the “exposure-outcome” relationship; ③ IVs should solely impact outcome variables through the exposure factors. Using a significance threshold of *p* < 5 × 10^−8^ as the screening criterion, statistically significant SNP loci associated with female reproductive characteristics were selected as the instrumental variables for preliminary screening. A linkage imbalance coefficient of r^2^ = 0.001 was employed, and the region width was set to 10000kb to ensure the independence of each SNP and mitigate the influence of gene polymorphism on the results. Additionally, SNPs closely associated with the results (*p* > 5 × 10^−8^) were excluded. Finally, the F-statistic was utilized to quantify the strength of the instrumental variables. A value greater than 10 suggests a reduced likelihood of weak instrumental bias in the included SNPs (Palmer et al., 2012). The analysis methods employed in this study were implemented using the “TwoSampleMR” and “MendelR” packages within R 4.2.3 software.

### 2.3 The UVMR and MMVMR

The random-effects inverse-variance-weighted (IVW) method exhibited the highest statistical power among all MR methods and was therefore selected as the primary analysis method for univariable Mendelian Randomization (UVMR) (Burgess et al., 2013). To mitigate potential bias in the IVW results arising from horizontal pleiotropy of individual SNPs, three additional MR methods were employed to enhance result robustness: MR-Egger, Weighted median, and Weighted mode. Additionally, we conducted multivariable Mendelian Randomization (MVMR) analysis, adjusting for EA and BMI, to evaluate the independent effects of AFS, AFB, AAM, ANM, and PA on frailty.

### 2.4 Sensitivity analysis

In UVMR analysis, the conventional IVW analysis methods may be susceptible to ineffective instrument bias or pleiotropy. Hence, this study aimed to assess the validity and robustness of the IVW results through sensitivity analysis. Firstly, the Cochran’s Q statistic is employed to assess the heterogeneity of SNPs. If the Cochran’s Q statistic test yields statistical significance, it indicates significant heterogeneity in the analysis results. Accordingly, if heterogeneity is present, a random-effects IVW model is employed; otherwise, a fixed-effects IVW model is utilized. To identify potential bias due to horizontal pleiotropy, an MR-Egger regression was employed. If the intercept term in the MR-Egger regression is not equal to 0 and is statistically significant (*p* < 0.05), it indicates the presence of horizontal pleiotropy in the study. Additionally, the Leave-one-out sensitivity test method is employed, which systematically removes each SNP one at a time. If the MR results obtained using the remaining SNPs do not significantly deviate from the overall results, it indicates the stability of the MR results.

Additionally, we conducted an MVMR study to evaluate the direct effect of female reproductive traits on frailty while controlling for BMI and EA. This control was necessary as obesity and EA could potentially have a confounding influence on the pathways linking female reproductive traits to frailty-related outcomes. In the MVMR analysis, we employed an extended version of the IVW-MR method known as MVMR-IVW. The selection of random effects or fixed effects was based on heterogeneity, following the approach described in UVMR. Sensitivity analysis was conducted using the MVMR-Egger and Weighted Median methods. The MVMR-Egger method accounts for both measured and unmeasured pleiotropy.

### 2.5 Mediation analysis

Traditional methods for analyzing mediating effects often require strict assumptions prior to their application. For instance, they assume the absence of unmeasured confounding between exposure factors, mediators, and outcomes (VanderWeele, 2016). Moreover, the presence of measurement errors in exposure or mediating variables can introduce substantial biases that cannot be disregarded (Blakely et al., 2013). However, mediation effect analysis based on the MR method can partially meet these hypothesis requirements. Randomly assigned genetic variants were utilized as IVs for the phenotype, thereby sufficiently stabilizing the effect estimates to account for confounding bias, reverse causation, and measurement error (Carter et al., 2021). In this study, a two-step MR analysis was conducted to elucidate the specific effects of BMI and EA in this pathway. It further examined the effects of BMI and EA on exposure factors and outcomes.

## 3 Results

### 3.1 The results of the UVMR analysis

**FIGURE 1:**
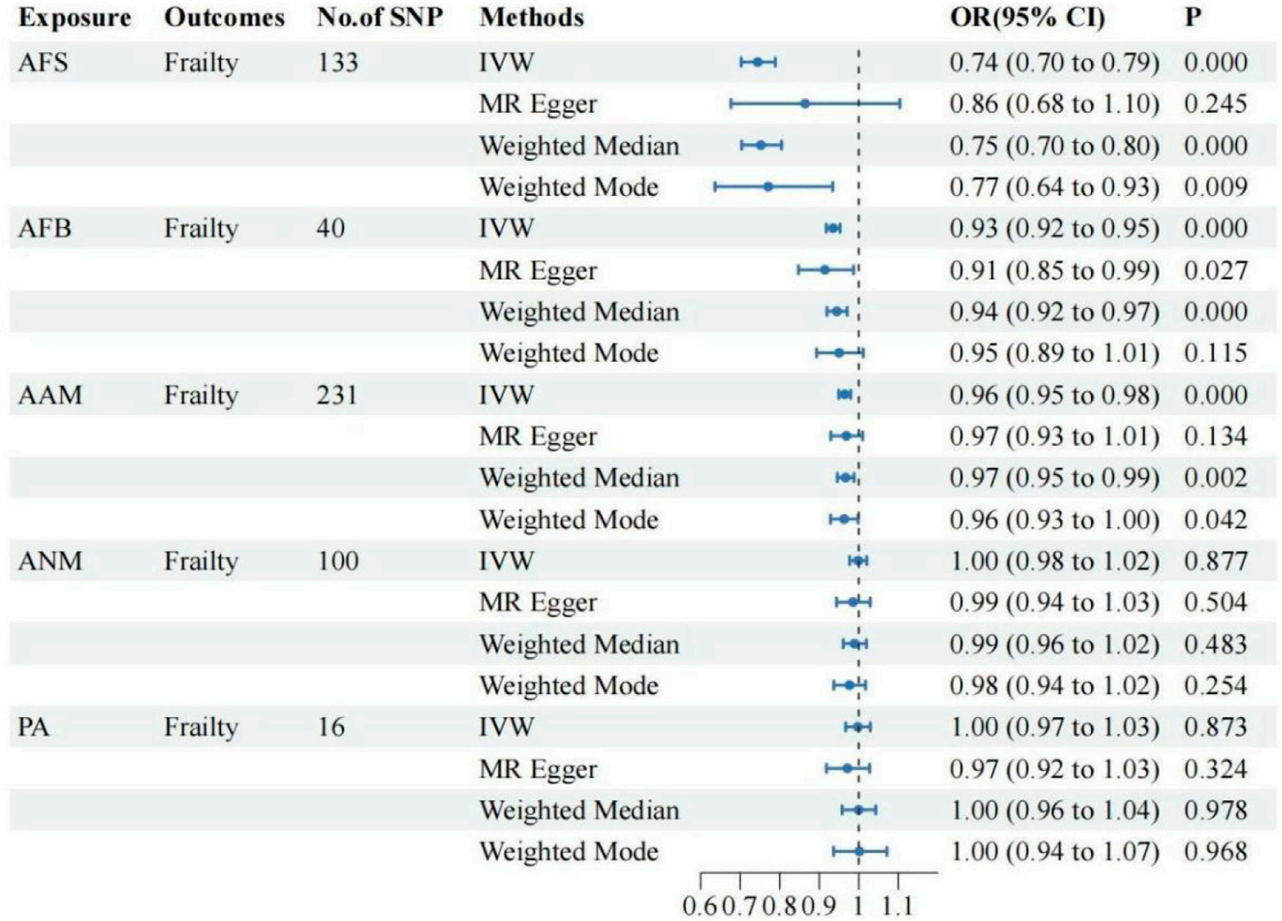
The UVMR results of the relationship between women’s reproductive factors and frailty.

**FIGURE 2:**
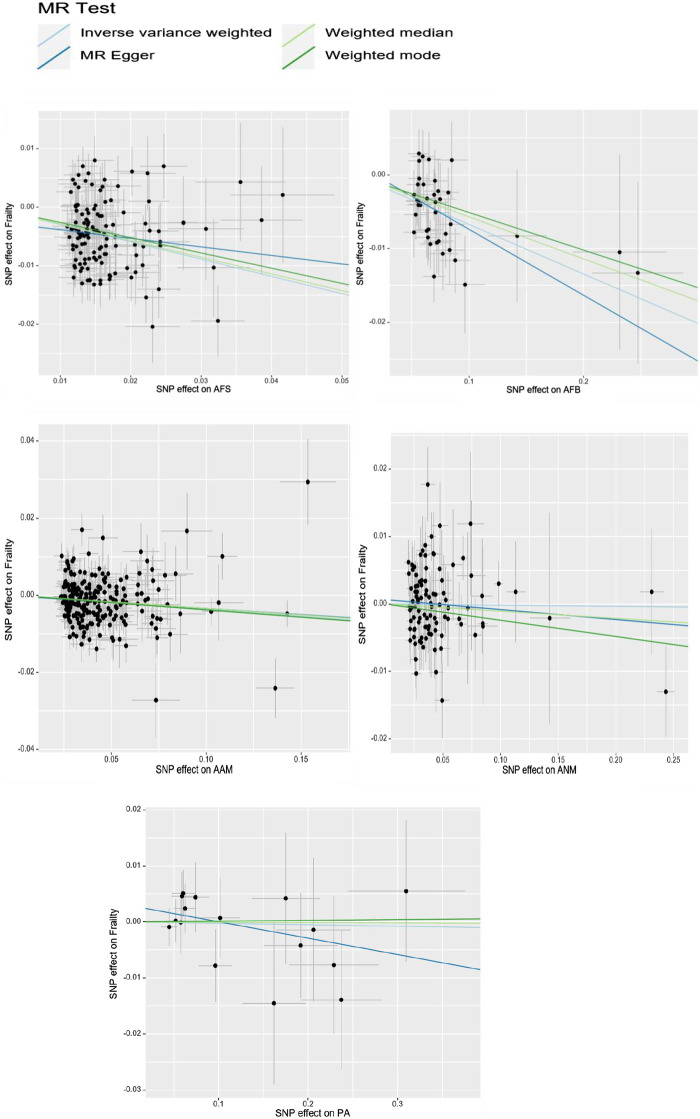
The scatter plot of UVMR of women’s reproductive factors and frailty.

The UVMR analysis, conducted at a significance level of α = 0.05, unveiled a causal correlation between genetically predicted female reproductive traits (AFS, AFB, and AAM) and the occurrence of frailty [IVW: OR = 0.74, 95%CI(0.70–0.79), *p* = 0.000; OR = 0.93, 95%CI(0.92–0.95), *p* = 0.000; OR = 0.96, 95%CI(0.95–0.98), *p* = 0.000]. Furthermore, the findings from MR-Egger, Weighted Median, and Weighted Mode analyses exhibited consistent patterns, despite the lack of statistical significance in the *p*-values. Nevertheless, ANM and PA did not exhibit a significant effect on frailty (*p* > 0.05), indicating the absence of a causal relationship. As shown in Figures 1, 2.

### 3.2 The results of the MVMR analysis

Following adjustment for BMI and EA, MVMR analysis was conducted separately on female reproductive traits and frailty, with a significance level set at α = 0.017.

**FIGURE 3:**
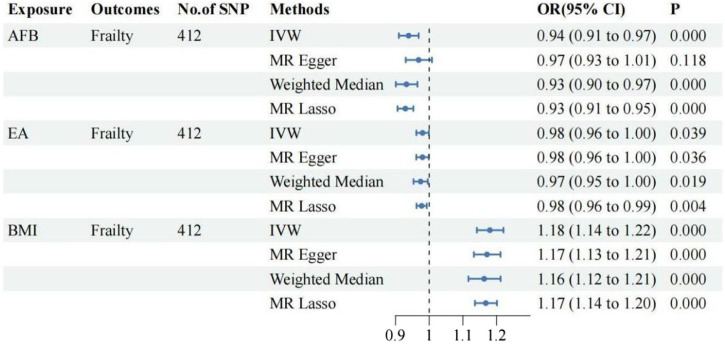
The relationship between AFB and frailty after adjusting for the effects of EA and BMI.

**FIGURE 4:**
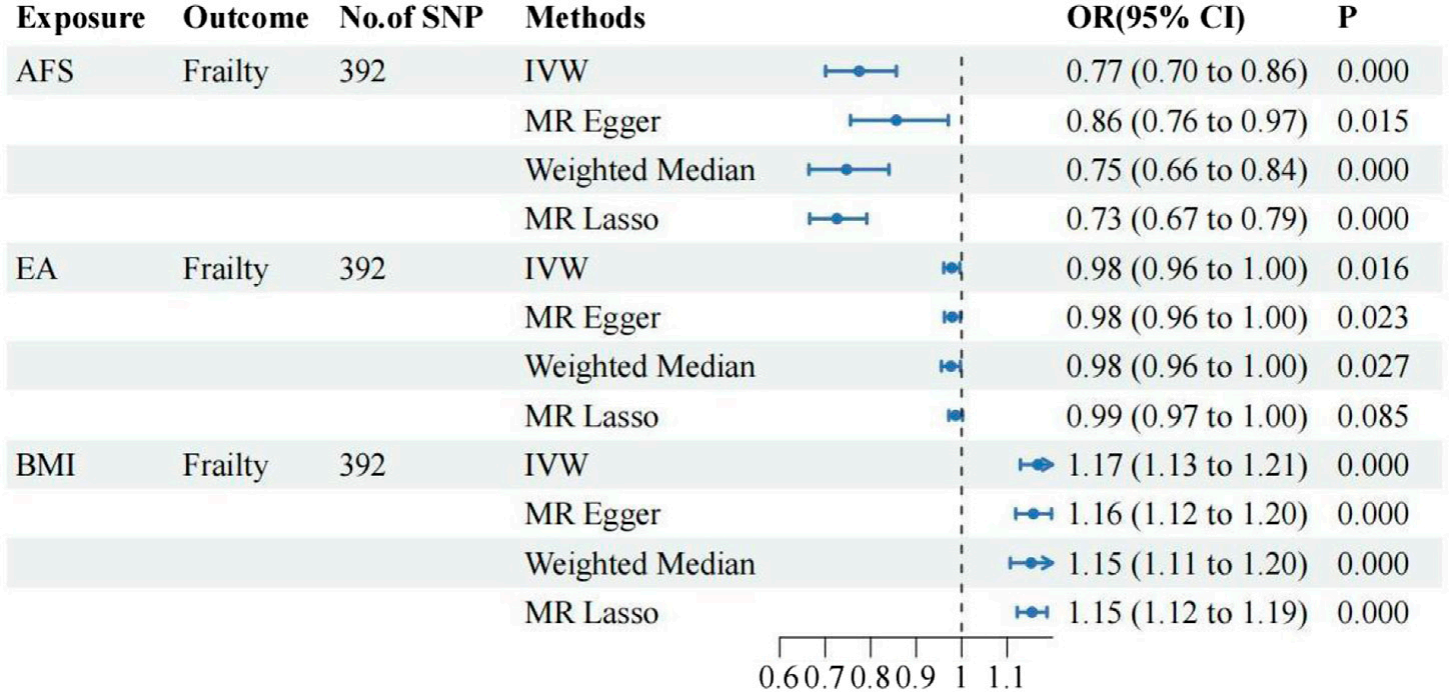
The relationship between AFS and frailty after adjusting for the effects of EA and BMI.

**FIGURE 5:**
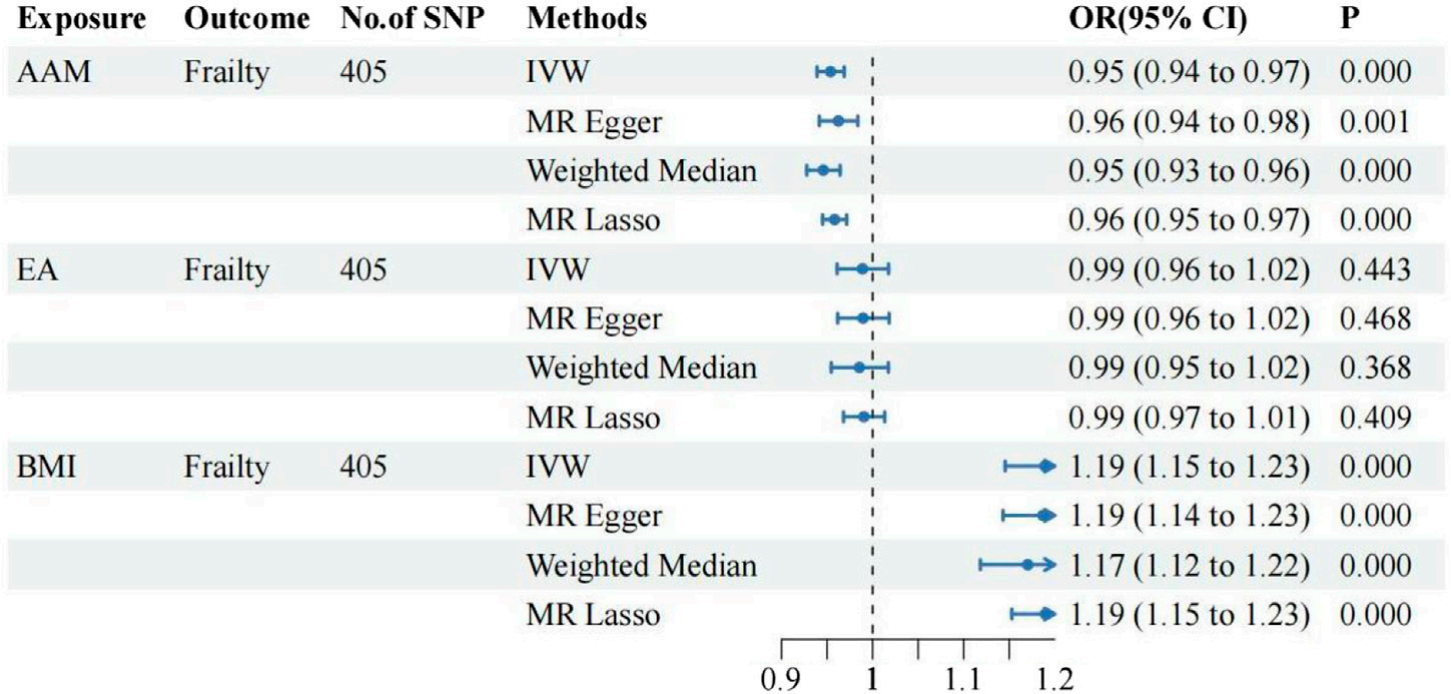
The relationship between AAM and frailty after adjusting for the effects of EA and BMI.

①Following adjustment for the effects of BMI and EA, the MVMR analysis revealed that AFB still had a significant effect on frailty [IVW: OR = 0.94, 95%CI(0.91–0.97), *p* = 0.000]. Additionally, MR-Egger, Weighted Median, and MR Lasso analyses exhibited similar trends, although the *p*-values of MR-Egger did not reach statistical significance. As shown in Figure 3; ②Following adjustment for the effects of BMI and EA, the MVMR analysis revealed that AFS still had a significant effect on frailty [IVW: OR = 0.77, 95%CI(0.70–0.86), *p* = 0.000]. Additionally, MR-Egger, Weighted Median, and MR Lasso analyses exhibited similar trends. As shown in Figure 4; ③Following adjustment for the effects of BMI and EA, the MVMR analysis revealed that AAM still had a significant effect on frailty [IVW: OR = 0.95, 95%CI(0.94–0.97), *p* = 0.000]. Additionally, consistent trends were observed in the results of MR-Egger, Weighted Median, and MR Lasso analyses. As shown in Figure 5.

### 3.3 Two-step MR analysis

BMI and EA may have a mediating role in the causal relationship between female reproductive traits (AFS, AFB, and AAM) and frailty. To analyze this mediation effect, a two-step MR analysis was employed.

①A two-step MR analysis was conducted to examine the mediation of BMI and EA in the relationship between AFB and frailty. The coefficient product method revealed that EA and BMI mediated 28.79% and 13.78% of the effect, respectively. Thus, EA and BMI emerged as the primary mediators in the causal relationship between AFB and frailty; ②A two-step MR analysis was performed to assess the mediation of BMI and EA in the relationship between AFS and frailty. According to the coefficient product method, the mediated ratios for EA and BMI were determined as 23.55% and 11.82%, respectively, establishing EA and BMI as the primary mediators in the causal association between AFS and frailty; ③A two-step MR analysis was conducted to examine the mediation of BMI and EA in the relationship between AAM and frailty. The analysis revealed that EA did not serve as a mediating factor in the causal relationship between AAM and frailty, indicating no mediation effect. However, according to the coefficient product method, BMI emerged as the primary mediator with a mediated ratio of 30.32% in the causal association between AAM and frailty. Detailed mediation MR analysis and effect results are shown in Table 2, 3.

**TABLE 2:** The causal relationship between exposure and outcome, exposure and mediation, mediation and outcome.

Exposure	Outcome	No.of SNP	Methods	OR	or_lci95	or uci95	
**AFB**	**Frailty**	**40**	**1VW**	**0935**	**0918**	**0.952**	**1.25E-12**
AFB	BMI	17	11.7W	0.955	0.940	0.969	3.29E-09
BMI	Frailty	470	IVW	1.221	1.189	1.254	6.13E-50
AFB	EA	34	IVW	1.510	1.388	1.642	6.76E-22
EA	Frailty	205	IVW	0.954	0.945	0.963	1.60E-23
**AFS**	**Frailty**	**133**	**1VW**	**0.744**	**0.702**	**0.789**	**1.71E-23**
AFS	BMI	42	IVW	0.540	0.787	0.896	1.31E-07
BMI	Frailty	470	IVW	1 221	1.189	1.254	6.13E-50
AFS	EA	93	IVW	4.359	3.592	5.362	1.80E-47
EA	Frailty	205	IVW	0.954	0.945	0.963	1.60E-23
**AAM**	**Frailty**	**231**	**1VW**	**0.964**	**0.949**	**0.979**	**541E-06**
AAM	BMI	76	IVW	0.946	0.926	0.967	4.22E-07
BMI	Frailty	470	IVW	1.221	1.189	1.254	6.13E-50
AAM	EA	227	IVW	1.053	0.996	1.114	0.069
EA	Frailty	205	IVW	0.954	0.945	0.963	1.60E-23

**TABLE 3:** The TE, DE and IE mediated by BMI and EA.

Exposure	Mediator	Outcome	TE Effect size (95% CI)	DE Effect size (95% C	IE Effect size (95% Cl)	Mediation effect proportion% (95% Cl)
AFB	EA	Frailty	−0.067 (0.086,-0.049)	−0.048 (-0 067,-0.029)	−0.019 (-0.025,-0.014)	28.79 (28.50,28.96)
AFB	BMI	Frailty	−0.067 (0.086,-0.049)	−0.058 (0.077,-0.039)	−0.009 (-0.013,-0.006)	13.78 (12.23,14.66)
AFS	EA	Frailty	−0295 (0353,-0.237)	−0.226 (0.286,-0.165)	−0.070 (-0.086,-0.053)	23.55 (22.31,24.39)
AFS	BMI	Frailty	−0295 (0353,-0237)	−0.260 (0320,-0.201)	−0.035 (-0.049,-0.021)	11.82 (8.90,13.78)
AAM	EA	Frailty	non-significant	−0.034 (-0.050,-0.018)	non-significant	non-significant
AAM	BMI	Frailty	0.036 (-0.052,-0.021)	−0.025 (-0.042,-0.009)	−0.011 (-0.016,-0.007)	30.32 (29.88,31.43)

Note: TE, total effect; DE, direct effect; IE, intermediary effect.

### 3.4 Sensitivity analysis results

**FIGURE 6:**
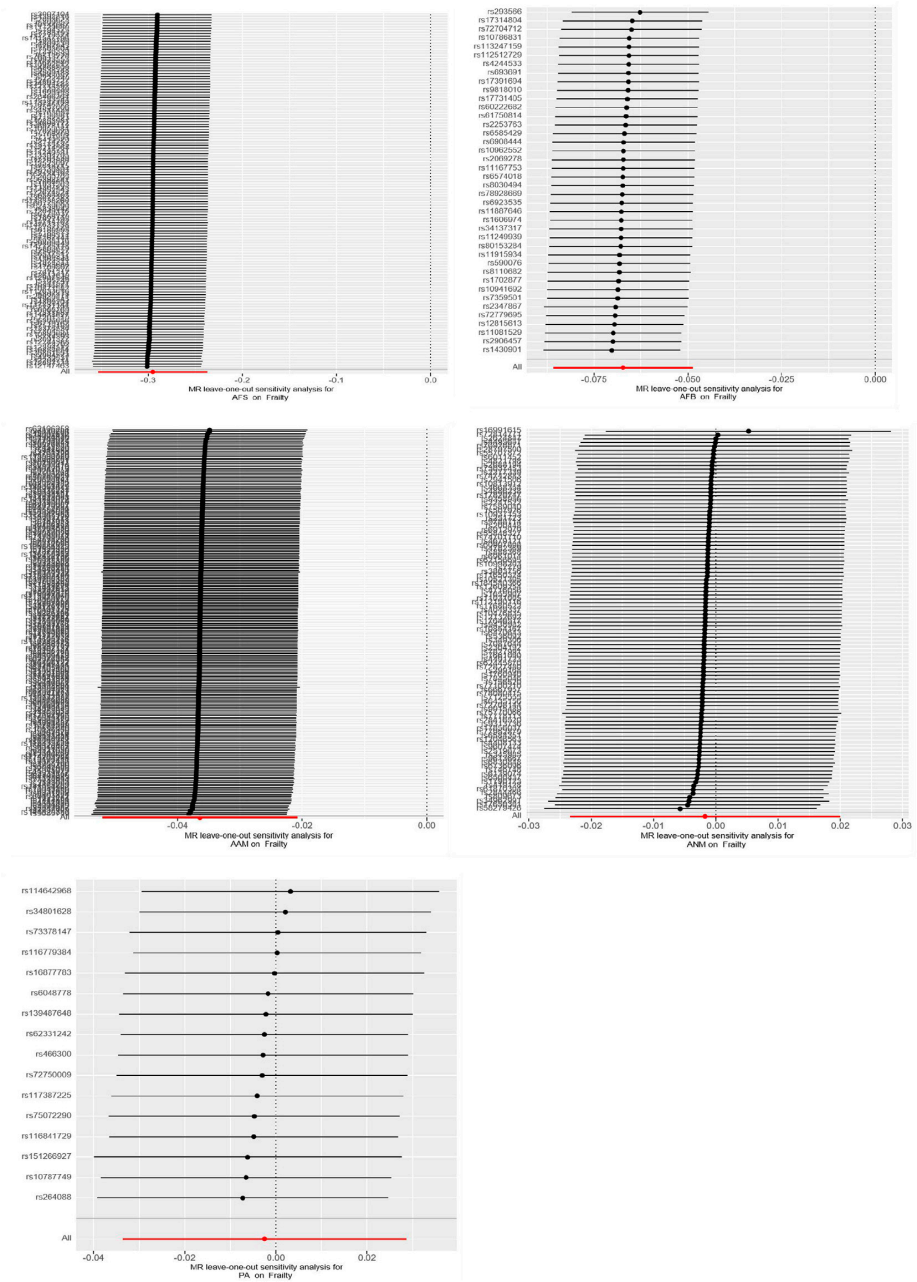
Heterogeneity of MR results from the sensitivity test of “Leave-one-out”.

Cochran’s Q tests were conducted for the UVMR analyses utilizing the MR-Egger and IVW methods. A *p*-value greater than 0.05 suggests no heterogeneity in the relationship between AFB and PA with frailty, while a *p*-value less than 0.05 indicates heterogeneity in the relationship between AFS, AAM, ANM, and frailty. Hence, the primary statistical analysis method employed is the IVW method (random effects model). The intercept terms of the MR-Egger regression did not provide evidence of horizontal pleiotropy. Furthermore, employing a Leave-one-out sensitivity analysis to assess the impact of each SNP site on overall causality, the results indicate no significant differences in the occurrence of the aforementioned causal relationships when systematically removing individual SNPs and repeating the MR analysis. This underscores the stability of the estimated effect results. In the MVMR analysis, employing Cochran’s Q test with the IVW method, all three groups of multivariate Mendelian analysis exhibited a *p*-value less than 0.017, indicating heterogeneity. However, in the analyses of AAM, EA, and BMI, the MR-Egger regression yielded a *p*-value greater than 0.05, suggesting no horizontal pleiotropy. See shown in Table 4; Figure 6.

**TABLE 4:** The sensitivity analysis results.

Exposure	Outcome	Heterogeneity	Pleiotropy test		
		IVW	MR Egger		
		Q	P	egger_intercept	P
**UVMR**					
**AFS**	**Frailty**	261519	<0.001	−0.002	0.220
**AFB**	**Frailty**	41351	0.368	0.002	0.558
**AAM**	**Frailty**	411.281	<0.001	−0.000	0.818
**ANM**	**Frailty**	135.006	0.009	0.001	0.494
**PA**	**Frailty**	8.097	0.920	0.003	0.280
**MVMR.**					
**AFB,EA,BMI**	**Frailty**	745.701	<0.001	−0.001	0.016
**AFS,EA,BMI**	**Frailty**	712.454	<0.001	−0.001	0.010
**AAM,EA,BMI**	**Frailty**	715.839	<0.001	−0.000	0.253

Note: AAM, age at menarche; AFB, age at first birth; AFS, age at first sexual intercourse; ANM, age at natural menopause;PA, pregnancy abortion; BMI, body mass index; EA, educational attainment; IVW, inverse-variance-weighted; UVMR, univariable mendelian randomization; MVMR, multivariable mendelian randomization.

## 4 Discussion

This study investigated the causal relationship between five female reproductive traits (AAM, ANM, AFB, AFS, and PA) and the risk of developing frailty. The GWAS summary statistics publicly available in the European population were utilized. The UVMR analysis demonstrated a causal relationship between AAM, AFB, and AFS, and the occurrence of frailty. Moreover, the analysis revealed that larger values of AAM, AFB, and AFS were associated with a lower risk of frailty. However, no causal relationship between ANM and PA with frailty was observed. The sensitivity analysis conducted using other MR analysis methods in the aforementioned results demonstrated robustness and revealed no evidence of horizontal pleiotropy, thereby supporting the reliability of our findings. MVMR analysis indicates that the aforementioned causal relationship remains significant, possibly attributed to the influence of BMI and EA. The Two-step MR analysis revealed that EA and BMI were the primary mediating factors in the causal relationship between AFB, AFS, and frailty. Additionally, BMI emerged as the main mediating factor in the causal relationship between AAM and frailty.

Epidemiology traditionally does not emphasize the investigation of the causal relationship between female reproductive traits and the occurrence of frailty. Therefore, this study represents the first attempt to explore this relationship from a genetic perspective. The risk of aging in the elderly is influenced by multiple factors, with the endocrine system playing a significant role. Female reproductive characteristics are closely associated with changes in estrogen levels. Studies have suggested that decreased estrogen levels may negatively impact skeletal muscle mass (Messier et al., 2011). Iannuzzi-Sucich et al. discovered a strong positive correlation between muscle mass and plasma estrogen levels in women (Iannuzzi-Sucich et al., 2002). Another researcher highlighted the direct impact of estrogen on muscle mass, attributing it to the presence of estrogen β receptors on the cell membrane, cytoplasm, and nuclear membrane of skeletal muscle cells. A decrease in estrogen levels may directly contribute to reduced protein synthesis in skeletal muscle (Brown, 2008). Additionally, BMI and EA may potentially influence the development of female reproductive characteristics, thereby exerting a mediating effect on frailty occurrence. In summary, our findings highlight a causal relationship between female reproductive traits (AFB, AFS, and AAM) and the risk of frailty. However, further investigation is needed to understand the detailed underlying mechanisms.

Our study has made significant advancements. The primary strength of our study lies in its proposition of an MR framework for genetically assessing the association between female reproductive traits and frailty. Additionally, it evaluates the proportion of potential mediators of various risk factors in this relationship. This study offers comprehensive and robust evidence to facilitate further exploration of the mechanisms underlying female reproductive characteristics and the pathogenesis of frailty. However, traditional observational studies are often plagued by residual confounding and reverse causality. Moreover, this study employed the most extensive GWAS summary data available to date for the analysis. Female reproductive traits were exclusively evaluated in females, whereas AFS, AFB, and frailty were assessed in both males and females, ensuring potential genetic variation effects are comparable between the sexes.

However, the heterogeneity observed in the study can be attributed to the following reasons. Firstly, AAM only included women, whereas frailty were tested in both men and women. Therefore, if the effects of the genetic variations differ between the two sexes, our results might be biased. Secondly, reproductive traits are complex and heterogeneous, resulting from a combination of genetic and environmental factors. The phenotypic variation in these traits cannot be solely attributed to genetics. For instance, AFB and AFS are primarily influenced by psychosocial, cultural, and economic factors, suggesting that genetic effects are likely intertwined with these factors. Additionally, the assessment of specific timing of reproductive characteristics relies primarily on questionnaires. The imprecise measurement of these factors can introduce measurement bias, thus we cannot exclude the possibility of bias resulting from inaccurate recall in previous studies (Mishra et al., 2021). Lastly, inadequate control of confounding factors, such as BMI and EA, which can impact both the exposure and outcome, may introduce bias in observational studies.

This study has several limitations that should be considered. Firstly, partial overlap between the samples used for exposure and outcome assessment may introduce bias in the results (Burgess et al., 2016), which may reduce the credibility of the results. In this regard, the strong strength of genetic instruments (all mean F > 10) and low degree of overlap (<10%) suggested considerable bias would not be expected (Pierce and Burgess, 2013). Secondly, this study utilized GWAS data from Europeans, and therefore, caution should be exercised when generalizing the results to other populations due to genetic variations. Future studies from diverse populations are necessary to validate our findings. Thirdly, MR analysis is limited to exploring causality and does not provide insights into specific biological mechanisms. Lastly, the GWAS data used in this analysis lacked stratified analysis results, such as sex, age, and disease course, our results may be subject to bias, and further investigation into sex-specific differences, disease course, etc., is limited.

## 5 Conclusion

In conclusion, this study presents compelling genetic evidence supporting a causal relationship between female reproductive traits (AFB, AFS, and AAM) and frailty, indicating that a younger age is associated with a higher risk of frailty. However, additional research is necessary to elucidate the underlying biological mechanisms. Considering the substantial impact of frailty on individuals and families, our findings underscore the significance of providing appropriate guidance on AFS and AFB, as well as monitoring AAM, EA, and BMI, in order to mitigate the risk of frailty.

## Data Availability

The datasets presented in this study can be found in online repositories. The names of the repository/repositories and accession number(s) can be found in the article/Supplementary Material.

## Abbreviations

AAM, Age at menarche; AFB, Age at first birth; AFS, Age at first sexual intercourse; ANM, Age at natural menopause; PA, Pregnancy abortion; BMI, Body mass index; EA, Educational attainment; MR, Mendelian randomization; UVMR, Univariable mendelian randomization; MVMR, Multivariable mendelian randomization; IVW, inverse-variance-weighted; IVs, instrumental variables; GWAS, Genome-Wide Association Study.
